# Antifungal Potential of Phytochemicals against *Mauginiella scaettae*, the Plant Pathogen Causing Inflorescence Rot of Date Palm

**DOI:** 10.1155/2021/1896015

**Published:** 2021-03-06

**Authors:** Eimad Dine Tariq Bouhlali, Mgal Derouich, Reda Meziani, Adil Essarioui

**Affiliations:** ^1^National Institute for Agricultural Research, Regional Center of Errachidia, Errachidia, Morocco; ^2^Biology,Environment and Health Team, Faculty of Sciences and Techniques, Moulay Ismail University, Errachidia, Morocco

## Abstract

Date palm (*Phoenix dactylifera* L.) inflorescence rot caused by *Mauginiella scaettae* poses a serious threat to date palm in Morocco. The present study aims to determine the antifungal activity of five plant extracts against *M. scaettae*, including *Acacia cyanophylla*, *Cupressus atlantica*, *Eucalyptus torquata*, *Nerium oleander*, and *Schinus molle* and link this effect to their content in phenolics and flavonoids, as well as their antioxidant properties. Plant extracts exhibited significant discrepancies regarding their antifungal activity (*p* < 0.05). The extracts of *E. torquata* and *C. atlantica* had the strongest and dose-dependent manner inhibitory effect against mycelial growth and spore germination. *E. torquata* and *S. molle* caused the greatest sporulation reductions of about 88.05% and 36.11%, respectively. In addition, there were significant differences among the examined plant extracts with respect to their total polyphenols (14.52–76.68 mg GAE/g DW), flavonoids (8.75–57.78 g RE/100 g DW), and antioxidant properties as measured by TEAC (74.77–391.23 mmol TE/g DW) and FRAP assays (87.18–474.04 mmol TE/g DW). Strong correlations were found between phenolic compounds and antioxidant activity suggesting that polyphenols play a key role in the observed antioxidant and antifungal activities.

## 1. Introduction

Date palm (*Phoenix dactylifera* L.) has long been a primordial fruit crop in North Africa and the Middle East [1]. Beside its fundamental ecological roles in creating a suitable microclimate for growing other crops, date palm constitutes a significant source of income and offers tremendous employment opportunities to local populations helping, thereby, in preventing rural exodus. [2].

Date palm in Morocco is cultivated over about 60 000 ha. These include 17 000 ha of modern plantations where valuable varieties, principally “Majhoul,” are grown using intensive management practices [3]. This system of massive production coupled with genetic homogeneity is a precursor to increased susceptibility to plant pathogenic agents. Inflorescence rot (also named “Khamedj” in North Africa) is one of the most prevalent diseases on date palm. It was first reported in Libya and attributed to the fungal pathogen *Mauginiella scaettae* Cav. though other fungal species such as *Thielaviopsis paradoxa, Fusarium moniliform*, *Fusarium oxysporum*, and *Fusarium solani* were found in rotted date palm inflorescences as well [4]. Inflorescence blight is most severe on poorly maintained palms in areas with prolonged winters and rainy springs [4]. Losses due to this disease range generally from 2 to 15%, but can occasionally exceed 50% in extreme epidemic cases [4, 5]. The management of the disease reposes primarily on sanitation practices and, ultimately, fungicide spray. In fact, several fungicides have been reported to be effective against this disease [4, 6]. However, because of their environmental impacts related to toxicological and ecotoxicological risks and owing to resistance build up in pathogen populations, there has been a growing interest in switching from the use of pesticide to environmentally friendly means of disease control, especially in organic farming settings [7–9]. Thus, there is a need to investigate new approaches for effective and safe control of inflorescence rot and ecologically respectful date production in Moroccan oases. ‬‬‬‬‬‬‬‬‬‬‬‬‬‬‬‬‬‬‬‬‬

Plants biosynthesize a wide range of phytochemicals including polyphenols, terpenoids, alkaloids, quinones, flavonoids, tannins, and steroids [10]. These compounds have been reported to enhance the plant defense system against pathogen attacks during which their concentration in plants depends on the pathosystem and environmental conditions [11]. Previous studies showed that plant-derived compounds from members of the genera of *Nerium*, *Schinus*, *Eucalyptus*, *Cupressus*, and *Acacia* possess potent antifungal activities against a broad spectrum of phytopathogenic fungi including *Fusarium oxysporum* f. sp. *albedinis*, the causal agent of Bayoud disease on date palm, and these properties were attributed to the antioxidant and the richness of these plants in secondary metabolites such as polyphenols and flavonoids [1, 8, 12–14]. These findings suggest potentially similar effects on *M. scaettae*, but data related to this topic are limited.

The objectives of this work are (i) to investigate antifungal effects of aqueous extracts of five plant species (*Nerium oleander, Eucalyptus torquata, Schinus molle, Cupressus atlantica,* and *Acacia cyanophylla*) against *M. scaettae*, (ii) to define their richness in secondary metabolites (polyphenols and flavonoids), and (iii) to measure their antioxidant properties. This work allows the construction of a more comprehensive understanding of mechanisms by which plant compounds may affect the survival and development of *M. scaettae*.

## 2. Materials and Methods

### 2.1. Plant Materials

The leaves, stems, twigs, and other parts of five plants, namely, *Nerium oleander*, *Eucalyptus torquata*, *Cupressus atlantica*, *Acacia cyanophylla*, and *Schinus molle*, were collected during November 2020 as wastes produced during green urban space management in Errachidia city (southeast of Morocco). The identification of these plant species was performed by Dr. Homrani Bakkali, a botanist in the National Institute of Agricultural Research, and the specimen of each species was prepared and placed in the herbarium of the same institute. The collected plant material was dried under shade, blended into powder using an electric grinder, and then, conserved at ambient temperature (25°C) away from light until manipulation.

### 2.2. Preparation of Rich Polyphenol Extracts

For each plant species, a stock solution (10%, w/v) was constructed following the method reported by Hmidani et al. [15] with slight changes. Summarily, thirty grams of plant powder was dissolved in 300 mL of 50°C water by means of a shaker incubator for six hours. The upcoming solutions were analyzed for antifungal and antioxidant powers.

### 2.3. Antifungal Activity

#### 2.3.1. Mycelial Growth Inhibition Assay

The ability of plant extracts to inhibit mycelial growth was evaluated according to the protocol established by Bouhlali et al. [1]. 5 mm mycelial discs from a 7-day-old culture of *M. scaettae* were transferred onto Petri dishes containing a PDA medium supplemented with different volumes of plant extracts sterilized through 0.2 *μ*m millipore filters to obtain 1%, 2%, 3%, and 4% serial dilutions. Thereafter, the plates were incubated at 25 ± 2°C for 14 days. Mycelial growth was determined as the average diameter of the colony measured at two right angles. Four replicates per treatment (plant extract ×  concentration) were performed with a negative control containing unsupplemented media. The percentage inhibition of mycelial growth (IP) was calculated using the following formula:

(1) $$\begin{array}{c} \text{Growth inhibition}\left(\%\right)=\frac{\left(D_{c}-D_{t}\right)\times 100}{D_{t}}, \end{array}$$

where *D*_*c*_: diameter of the colony in the control (mm) and *D*_*t*_: diameter of the colony in the presence of the plant extract (mm).

#### 2.3.2. Spore Germination Inhibition Assay

The spore germination inhibition was assessed following the method described by Bammou et al. [16]. Briefly, an aliquot (100 *µ*L) of *M. scaettae* spore suspension adjusted to 10^3^ spores/mL of sterile distilled water (by means of a Malassez hemocytometer) was spread over the Petri dishes containing the PDA medium supplemented with plant extracts as indicated above. The number of spores germinating out of 100 counted was determined after 24 hours of incubation at 25 ± 2°C. A spore was considered as germinating when the length of its germ tube was greater than its smallest diameter. Each treatment included four replicates and a negative control containing unsupplemented media. The percentage of spore germination inhibition (PSGI) by each plant extract was calculated using the following equation:

(2) $$\begin{array}{c} \text{Inhibition of spore germination }\left(\%\right)=\frac{\left(N_{c}-N_{t}\right)\times 100}{N}, \end{array}$$

where *N*_*c*_: number of germinating spores in the control and *N*_*t*_: number of germinating spores in the presence of the plant extract.

#### 2.3.3. Sporulation Inhibition Assay

The plates used to perform the mycelial growth inhibition assay were kept in incubation for another day, under the same conditions, to help assess the effect of plant extracts on *M. scaettae* sporulation according to the method described by Bouhlali et al. [4]. For each colony, four discs (5 mm) taken along a diameter were transferred into a 1 mL tube of sterile distilled water. The tube was vortexed for 30 seconds, and the spore concentration of the resulting suspension was determined using a Malassez cell chamber. Four counts per suspension were made, and the values were expressed as the number of spores per unit area (mm^2^). The percentage of sporulation inhibition (SI) was determined by means of the following formula:

(3) $$\begin{array}{c} \text{Sporulation inhibition}\left(\%\right)=\frac{\left(N_{c}-N_{t}\right)\times 100}{N}, \end{array}$$

where *N*_*c*_: number of spores estimated in the control and *N*_*t*_: number of spores estimated in the presence of the plant extract.

### 2.4. Antioxidant Activity

#### 2.4.1. Measurement of the Total Phenolic Content

The total phenolic content (TPC) in each plant extract was measured following the method described by Derouich et al. [17]. Briefly, a hundred microliter of the diluted plant filtrate was mixed with 500 *µ*L of 1/10 water-diluted Folin–Ciocalteau's reagent. Subsequently, 400 *µ*L of sodium carbonate solution (7.5% *w/v*) was added. The mixture was left at room temperature for 60 min, and then, the absorbance was read at 765 nm. Gallic acid was used to prepare the calibration curve which stated the concentration range of the Gallic acid standard solutions (0–500 mg/L). The results (means of four measurements) were expressed in mg Gallic acid equivalents per gram dry weight (DW) of plant material (mg GAE/g DW).

#### 2.4.2. Measurement of the Total Flavonoid Content

The total flavonoid content (TFC) of plant material was measured according to the method previously described by Bouhlali et al. [1]. A sample of five hundred microliters of diluted plant filtrate was filled up to 2500 *µ*L with distilled water, mixed with 150 *µ*L of 5% sodium nitrite and 10% aluminum chloride, and then, incubated for 5 min at ambient temperature before adding 1 mL of 1 M sodium hydroxide. The final volume of the mixture was made up to 5000 *µ*L using distilled water. The absorbance of the resulting solution was measured at 510 nm after homogenization. The absorbance was calibrated to a standard curve prepared using Rutin at various concentrations ranging from 0 to 800 mg/L. The results (means of four measurements) were expressed in mg Rutin equivalents (RE) per gram DW of plant material (mg RE/g DW).

#### 2.4.3. Trolox Equivalent Antioxidant Capacity (TEAC)

The TEAC assay was performed using the method of Re et al. [18]. Aqueous solutions of both ABTS (2,2′-azino-bis (3-ethylbenzothiazoline-6-sulfonic acid)) (7 mM) and potassium persulphate (2.45 mM) were mixed and kept in the dark at room temperature for 12–16 hours to generate ABTS radical cations (ABTS+). Subsequently, the absorbance of the mixture at 734 nm was adjusted to 0.700 ± 0.005 by adding distilled water. Finally, 30 *μ*L of diluted plant filtrate was added to 3 mL of the diluted ABTS radical solution. The mixture was allowed to sit for 6 min at room temperature before the absorbance was measured at 734 nm. The total antioxidant activity (mean of four measurements) was evaluated in mmol of trolox equivalent per gram DW (mmol TE/g DW) using an aqueous solution of trolox as a standard curve.

#### 2.4.4. Ferric-Reducing Antioxidant Power Assay (FRAP)

The ferric-reducing ability of plant extracts was assessed according to the method previously described by Derouich et al. [19]. Briefly, 5 mL of TPTZ (2,4,6-tripyridyl-s-triazine) solution (10 mM TPTZ in 40 mM HCl), 50 mL of acetate buffer (300 mM, pH 3.6), and 5 mL of FeCl3 (20 mM in water solution) were mixed to prepare the FRAP reagent. For each sample, 10 *µ*L of plant filtrate was added to 2 mL of the FRAP reagent. The resulting solution was incubated at room temperature for 30 min before the absorbance at 593 nm was measured against a blank solution. Total antioxidant activity (mean of four measurements) was expressed in mmol of trolox equivalent per gram DW (mmol TE/g DW) using an aqueous solution of trolox as a standard curve.

### 2.5. Statistical Analysis

The statistical analysis was performed using SPSS version 23 software. One-way analysis of variance (ANOVA) and *post hoc* Bonferroni tests were used to determine significant differences between plant extracts in respect to their antifungal and antioxidant properties, as well as phenolic and flavonoid contents with *p* < 0.05 as the significance level. In addition, Pearson's square correlation coefficient (*R*^2^) was calculated to measure pairwise associations among variables.

## 3. Results

### 3.1. Antifungal Activity

**Figure 1:**
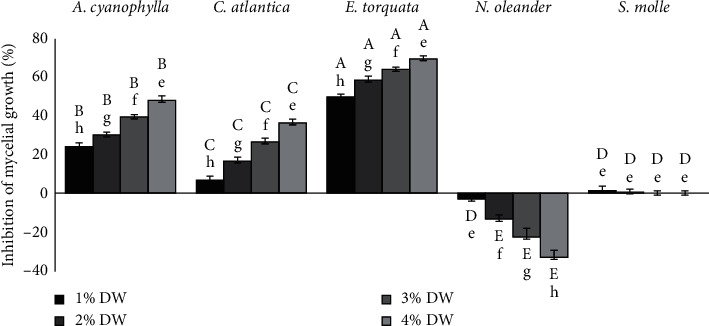
Effect of different plant DW percentages on *M. scaettae* mycelial growth. Plant species and extract concentration had significant effects on the percentage of radial growth inhibition (one-way ANOVA,*p* < 0.05). Each bar represents the mean of four replicate plates per concentration. Within each plant species, bars followed by the same lowercase letters indicate no significant differences among extract concentrations, while within each concentration, bars followed by the same uppercase letters indicate no significant differences among plant species (Bonferroni tests, *p* < 0.05). Error bars represent standard errors.

The plant extracts and their concentrations had significant effects on mycelial growth, spore germination, and sporulation (*p* < 0.05). At each concentration, *E. torquata* had the greatest inhibitory effect on mycelial growth, followed by *A. cyanopylla*, *C. Atlantica*, and *S. molle* (Figure 1). In‬ addition, ‬higher‬ plant‬ extract's‬ concentration‬ induced‬ more‬ potent inhibition ‬of‬ the‬ pathogen's ‬growth ‬except ‬for‬ *S. molle* which recorded a low inhibition of mycelial growth. In the PDA medium containing 1% and 4% of plant extracts, growth promotion rates of *N. oleander* extract were 3.06% and 33.12%, respectively.

**Figure 2:**
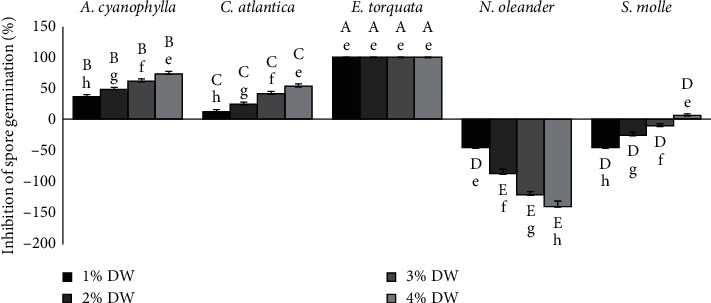
Effect of different plant DW percentages on *M. scaettae* spore germination. Plant species and extract concentration had significant effects on the percentage of inhibition of spore germination (One-way ANOVA, *p* < 0.05). Each bar represents the mean of four replicate plates per concentration. Within each plant species, bars followed by the same lowercase letters indicate no significant differences among extract concentrations, while within each concentration, bars followed by the same uppercase letters indicate no significant differences among plant species (Bonferroni tests, *p* < 0.05). Error bars represent standard errors.

As shown in Figure 2, the inhibition of spore germination was dose dependent for *A. cyanophylla* and *C. atlantica*. As for *N. oleander*, the increase in concentration promotes spore germination. However, the stimulation of spore germination is inversely proportional to the extract concentration for *S. molle*. At low concentration of extract (1%), *E. torquata* caused the strongest inhibition of spore germination (100%), followed by *A. cyanophylla* (73%), *C. atlantica* (53%), and *S. molle* (6%) at 4%, while the extract of *N. oleander* promoted spore germination by 141% at the highest dose of extract (4%).

**Figure 3:**
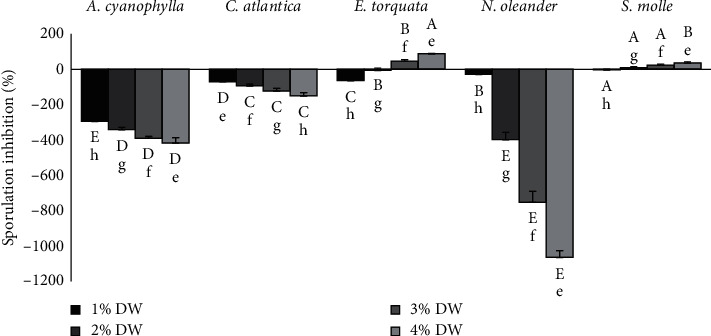
Effect of different percentages of plant DW on *M. scaettae* sporulation. Plant species and extract concentration had significant effects on the percentage of sporulation inhibition (one-way ANOVA, *p* < 0.05). Each bar represents the mean of four replicate plates per concentration. Within each plant species, bars followed by the same lowercase letters indicate no significant differences among extract concentrations, while within each concentration, bars followed by the same uppercase letters indicate no significant differences among plant species (Bonferroni tests, *p* < 0.05). Error bars represent standard errors.

Finally, plant extracts showed significant variations on sporulation of *M. scaettae*. In fact, *E. torquata* and *S. molle* extracts caused the greatest reduction in sporulation by 88.05% and 36.11% at 4%, respectively (Figure 3), while at low concentrations (≤2%), these species improved fungal sporulation. However, the other extracts promoted *M. scaettae* sporulation in a dose-dependent manner: *N. oleander* > *A. cyanophylla* > *C. atlantica*.

### 3.2. Polyphenol and Flavonoid Contents

The contents of polyphenols and flavonoids in plant extracts were significantly different (*p* < 0.05) and varied between 76.68 mg GAE/g DW in *E. torquata* and 14.52 GAE/g DW in *N. oleander*. Similarly, *E. torquata* extract recorded the highest content of flavonoids (57.78 mg RE/g DW), followed by *A. cyanophylla*, *C. atlantica*, *S. molle*, and *N. oleander* which showed the lowest content of flavonoids (8.75 mg RE/g DW).

### 3.3. Antioxidant Activity

The antioxidant property measured by the two methods FRAP and ABTS varied significantly (*p* < 0.05) (Table 1) and very strongly correlated with total polyphenol and flavonoid levels (*R*^2^ ≥ 0.98-*p* < 0.01) (Table 2). ABTS test values ranged from 74.77 to 391.23 mmol TE/g DW and were strongly correlated with FRAP test values ranging from 87.18 to 474.04 mmol TE/g DW (*R*^2^ = 0.99-*p* < 0.01). The highest antioxidant power for both assays was established by *E. torquata* extract, and the lowest was found in *N. oleander* extract.

Strong correlations were also concluded between polyphenol, flavonoid levels, and mycelial growth inhibition (*R*^2^ ≥ 0.91-*p* < 0.01), as well as spore germination (*R*^2^ ≥ 0.82, *p* < 0.01), while a low correlation was established between phenolic compounds and sporulation inhibition (*R*^2^ ≤ 0.33-*p* < 0.01).

## 4. Discussion

The management of date palm inflorescence rot is traditionally based on preventive cultural practices and, ultimately, fungicide sprays prior to full bloom [2]. In parallel, the growing demand on organically produced dates coupled with increasing environmental and health concerns over the use of pesticides have driven research for the development of alternative control strategies. Specifically, the use of biopesticides derived from such a wide range of natural materials as bacteria, fungi, viruses, nematodes, and plant extracts have been reported to hold great potential in alleviating plant disease stress [20]. For example, plant-derived compounds have been shown to act directly or indirectly against plant pathogens by inhibiting pathogen growth and/or inducing plant resistance [21]. These properties have put forward plant extracts as an important source of potentially antifungal active molecules for large-scale use against pathogenic fungi in agricultural settings. This study sheds more light on the capacity of plants to produce bioactive substances by evaluating the antifungal activity of the extracts of five plant species, *E. torquata*, *A. cyanophylla*, *S. molle*, *N. oleander*, and *C. atlantica*, toward *M. scaettae* and relating their biological effects to their antioxidant properties.

Our results provide strong evidence that *E. torquate* extract is highly effective in inhibiting sporulation, spore germination, and mycelial growth in *M. scaettae*. This implies that a potential exists for a biopesticide from *E. torquate* to hamper pathogen dissemination, infective capacity, and progression in the host. These results are consistent with those reported by Ameziane et al. [22] who found that *E. globulus* extract impedes completely the mycelial growth of *Geotrichum candidum* (also called *Geotrichum scaettae*), a plant pathogen of the same genus as the fungus under study. Additionally, our findings are in line with a large body of work that highlights the notoriety of members of the genus *Eucalyptus* as producers of fungicidal or fungistatic substances against a broad spectrum of plant pathogens, *A. Niger*, *A. flavus*, *A. parasiticus*, *G. lucidum*, *P. digitatum*, *A. alternata*, *A. solani*, *C. lunata*, *F. solani*, *F. moniliforme*, and *T. paradoxa* [13, 22–24]. Furthermore, *M. scaettae,* along with the fungal pathogens *F. oxysporum*, *T. paradoxa*, and *R. solani*, was also shown to be susceptible to the action of henna (*Lawsonia inermis*) extract [25], though this effect was slightly weaker than that in our investigation. Overall, these results lay the foundation for further studies on the use of extracts from *E. torquata* and, potentially, other plants, for the control of date palm inflorescence rot.

Although *A. cyanophylla* and *C. atlantica* exhibited strong inhibitory activities on mycelial growth and spore germination, their stimulating effects on sporulation raises concern about potential use in the control of *M. scaettae* as spore production plays a key role in dissemination and survival. In fact, under stressful conditions, mycelial growth is hampered and the fungus sporulates abundantly to ensure its persistence. In agreement with our finding, Dahlberg and Etten [26] have reported that sporulation generally occurs when the growth rate is reduced and delayed under conditions favoring rapid mycelial growth. The dose-dependent stimulation of sporulation by these plants may be due to their richness in tannins known for their ability to form complexes with vital metals such as iron, copper, and zinc and prevent their absorption by fungi [27]. Indeed, Su et al. [28] have reported that nutrient substrate depletion or lack of certain nutrients often stimulates sporulation. Several studies have reported that the type and concentration of carbon and nitrogen sources, as well as the *C*/*N* ratio, play important roles in fungal growth and sporulation [29, 30]. Therefore, the sugar composition and nitrogen content of *A. cyanophylla* and *C. atlantica*, as well as their phenolic composition, could be responsible for differential impact on mycelial growth, spore germination, and sporulation. Globally, because of their powerful effects on the mycelial growth, the extracts of *A. cyanophylla* and *C. atlantica* can be used to control inflorescence rot on date palm. However, their action should be completed by other antisporulation treatments.

Conversely, *N. oleander* promoted the pathogen's growth and development, suggesting that its extract contains growth-promoting substances such as minerals and vitamins, or provides preferred sources of organic carbon and nitrogen [31]. Consistent with these results, several studies have reported that PDA media supplemented with plant extracts improved growth and sporulation of various fungal pathogens [32, 33]. Regardless of the mechanism by which components from *N. oleander* support the growth of *M. scaettae,* this plant does not hold premise of using its extract in the management of date palm inflorescence blight.

There is a consensus that the antifungal effect of plants could be associated with the quantity and/or quality of their secondary metabolites [34]. Indeed, correlation analysis revealed that inhibition of mycelial growth and spore germination were very strongly correlated with polyphenol and flavonoid levels. As reported by El-Maati et al. [34], plant extracts with higher antimicrobial ability had higher phenolic content. Alternatively, Assiri et al. [35] have demonstrated that bioactive lipids including fatty acids and hydrophobic vitamins are implicated in the antimicrobial properties of plant extracts. All these compounds can work by inhibiting metabolic enzymes, interfering with cell wall synthesis and electron transport, altering cell permeability, inhibiting nutrient absorption, and interfering with other cellular metabolic pathways [36]. Strong correlations were also found between the antioxidant activity and inhibition of mycelial growth and spore germination (*R*^2^ ≥ 0.86; *p* < 0.01). In fact, antioxidants have been reported to play a major role in increasing the effectiveness of treatments against plant fungal pathogens when combined as adjuvants with fungicides [37]. Their effect may be due to an increase in membrane permeability, subsequently allowing a greater diffusion of fungicides in cells, or reduced oxidation of intracellular fungicides resulting in higher toxicity for fungi [38].

## 5. Conclusions

This study underlines the potential for using plants as a source of bioactive compounds for the control of inflorescence rot in date palm plantations. Our results showed that the extracts of *E. torquata*, *A. cyanophylla*, and *C. atlantica* are able to undermine growth of *M. scaettae*, the causal agent of this disease. The resulting effect of these plants can be attributed to their antioxidant polyphenols content and antioxidant properties. This implies possible use of measurements of these characteristics as a surrogate in the prediction of potential antimicrobial activities in plants. Globally, our work calls to attention the significance of further exploration of plants as an insufficiently tapped resource for the discovery and the exploitation of new molecules in the management of date palm pathogens.

## Figures and Tables

**Table 1:** Total antioxidant compounds and antioxidant activity of different plant extracts.

Plant species	TPC (mg GAE/g DW)	TFC (mg RE/g DW)	TEAC (mmol TE/g DW)	FRAP (mmol TE/g DW)
*A. cyanophylla*	58.60 ± 0.64^b^	47.82 ± 0.65^b^	284.42 ± 2.32^b^	365.20 ± 2.45^b^
*C. atlantica*	21.83 ± 0.35^c^	11.66 ± 0.42^c^	105.42 ± 2.04^c^	134.94 ± 1.88^c^
*E. torquata*	76.68 ± 0.69^a^	57.78 ± 0.61^a^	391.23 ± 5.88^a^	474.04 ± 2.49^a^
*N. oleander*	14.52 ± 0.29^e^	8.75 ± 0.67^e^	74.77 ± 1.57^e^	87.18 ± 0.92^e^
*S. molle*	18.31 ± 0.26^d^	8.91 ± 0.59^d^	93.13 ± 1.35^d^	112.78 ± 1.30^d^

TPC: total phenolic content, TFC: total flavonoid content, TEAC: trolox equivalent antioxidant capacity, and FRAP: ferric-reducing antioxidant power. Values are means ± standard deviation (SD). Averages, in the same column, followed by the same alphabetical letters are not significantly different according to *post hoc* Bonferroni test (*p* < 0.05).

**Table 2:** Correlation between phenolic compounds, antioxidant, and antifungal activities assays.

	TPC	TFC	FRAP	TEAC	PRGI	PISG	PSI
TPC	1						
TFC	0.98^*∗∗*^	1					
FRAP	1^*∗∗*^	0.99^*∗∗*^	1				
TEAC	0.99^*∗∗*^	0.98^*∗∗*^	0.99^*∗∗*^	1			
PRGI	0.94^*∗∗*^	0.94^*∗∗*^	0.94^*∗∗*^	0.95^*∗∗*^	1		
PISG	0.87^*∗∗*^	0.82^*∗∗*^	0.86^*∗∗*^	0.87^*∗∗*^	0.91^*∗∗*^	1	
PSI	0.26^*∗*^	0.33^*∗∗*^	0.26^*∗*^	0.22^*∗*^	0.23^*∗∗*^	0.34^*∗∗*^	1

TPC: total phenolic content; TFC: total flavonoids content; FRAP: ferric reducing antioxidant power; TEAC: trolox equivalent antioxidant capacity; PRGI: percentage of radial growth inhibition; PISG: percentage of inhibition of spore germination; PSI: percentage of sporulation inhibition, ^*∗∗*^*p* < 0.01 and ^*∗*^*p* < 0.05.

## Data Availability

No data were used to support this study.

## Abbreviations

TEAC: Trolox equivalent antioxidant capacity
FRAP: Ferric-reducing antioxidant power
TPTZ: 2,4,6-Tripyridyl-s-triazine
TE: Trolox equivalent
DW: Dry weight
GAE: Gallic acid equivalent
RE: Rutin equivalent
TPC: Total phenolic content
TFC: Total flavonoids content
PRGI: Percentage of radial growth inhibition
PISG: Percentage of inhibition of spore germination
PSI: Percentage of sporulation inhibition.
